# Characterization and Discrimination of Marigold Oleoresin from Different Origins Based on UPLC-QTOF-MS Combined Molecular Networking and Multivariate Statistical Analysis

**DOI:** 10.3390/metabo14040225

**Published:** 2024-04-15

**Authors:** Xingfu Cai, Juanjuan Wu, Yunhe Lian, Shuaiyao Yang, Qiang Xue, Dewang Li, Di Wu

**Affiliations:** 1 Chenguang Biological Technology Group Co., Ltd., Handan 057250, Chinalianyunheccgb@163.com (Y.L.);; 2 Key Laboratory of Comprehensive Utilization of Plant Resources in Hebei Province, Handan 057250, China; 3 Chenguang Biological Technology Group HanDan Co., Ltd., Handan 056000, China

**Keywords:** marigold, UPLC-QTOF-MS/MS, molecular networking, multivariate statistical analysis

## Abstract

Marigold oleoresin is an oil-soluble natural colorant mainly extracted from marigold flowers. Xinjiang of China, India, and Zambia of Africa are the three main production areas of marigold flowers. Therefore, this study utilized ultra-performance liquid chromatography-quadrupole time-of-flight mass spectrometry (UPLC-QTOF-MS/MS) technology, combined with Global Natural Products Social Molecular Networking (GNPS) and multivariate statistical analysis, for the qualitative and discriminant analysis of marigold oleoresin obtained from three different regions. Firstly, 83 compounds were identified in these marigold oleoresin samples. Furthermore, the results of a principal component analysis (PCA) and orthogonal partial least squares discriminant analysis (OPLS-DA) indicated significant differences in the chemical compositions of the marigold oleoresin samples from different regions. Finally, 12, 23, and 38 differential metabolites were, respectively, identified by comparing the marigold oleoresin from Africa with Xinjiang, Africa with India, and Xinjiang with India. In summary, these results can be used to distinguish marigold oleoresin samples from different regions, laying a solid foundation for further quality control and providing a theoretical basis for assessing its safety and nutritional aspects.

## 1. Introduction

Marigold oleoresin, also known as lutein oleoresin, is an oil-soluble natural colorant and antioxidant extracted from the flowers of *Tagetes erecta* L. It primarily contains lutein and lutein esters as its main active ingredients, which, together, constitute 70–79% of the total carotenoid content in marigold oleoresin [1]. Incorporating marigold oleoresin into food and nutritional supplements can effectively enhance the products’ antioxidant properties, protecting consumers against chronic diseases triggered by oxidative stress, such as cardiovascular disease, cancer, and neurodegenerative disorders. Additionally, lutein is particularly beneficial for eye health, helping to prevent macular degeneration and cataracts, thereby maintaining visual well-being [2]. In skincare and cosmetic products, adding marigold oleoresin helps to improve their antioxidant, moisturizing, and reparative functions, meeting consumer demands for natural, safe, and efficacious skincare solutions. In animal nutrition, the inclusion of marigold oleoresin not only increases the commercial value of poultry products, but also improves animal health and enhances farming efficiency [3]. In confectionery, beverages, baked goods, and seasonings, incorporating marigold oleoresin imparts attractive colors and supplementary nutrition. Furthermore, studies suggest that certain components in marigold oleoresin may exhibit anti-inflammatory, antimicrobial, antiviral, and anticancer bioactivities, demonstrating potential applications in the pharmaceutical field.

Marigold oleoresin, prepared by the hexane extraction of *Tagetes erecta* L., is the front-end raw material for producing lutein crystal and lutein ester. At present, the global marigold oleoresin market volume is about 10,000 tons, 80% of which is used as feed and 20% for health foods and pharmaceutical materials. The main producing areas of *Tagetes erecta* L. are China, India, and Africa, among which, about 600,000 mu is from China and about 300,000 mu is from India, while Zambia in Africa is now attempting to plant marigold due to its abundant planting area and suitable climate. However, the majority of existing studies have focused on lutein crystals and lutein ester, while there have been few studies on marigold oleoresin. Based on the above reasons, research on marigold oleoresin becomes very important and urgent. Therefore, marigold oleoresins from Xinjiang, India, and Africa were selected as research subjects in this study, with the aim of determining the chemical compositions and composition differences among marigold oleoresins from different regions.

Ultra-performance liquid chromatography coupled with time-of-flight mass spectrometry (UPLC-QTOF-MS/MS) is a highly sensitive and high-resolution platform extensively utilized for the identification of chemical components in herbal substances [4,5,6]. Global Natural Products Social Molecular Networking (GNPS) is a publicly accessible web platform (https://gnps.ucsd.edu accessed on 17 May 2023) that enables the systematic comparison and classification of numerous molecules based on the similarity of their MS/MS spectra. It also provides visual representations of the relationships among these molecules. GNPS is currently widely employed in metabolomics, the identification of chemical components in herbal medicines, and the discovery of novel compounds [7,8,9]. Furthermore, multivariate statistical analysis methods such as principal component analysis (PCA) and orthogonal partial least-squares-discriminant analysis (OPLS-DA) offer reliable approaches for assessing overall variation and identifying chemical markers for distinguishing the origin, different parts, and processing methods of herbs [10,11,12,13,14]. In summary, UPLC-QTOF-MS/MS, GNPS, and multivariate statistical analysis techniques play crucial roles in the identification and characterization of chemical components in herbal substances, facilitating the discovery of new compounds and the differentiation of herbal samples based on their origins and processing methods.

In this study, a comprehensive targeted metabolomic approach combining UPLC-QTOF-MS/MS with molecular networking (MN) was employed to perform a comparative analysis of metabolites in liposoluble extracts of marigold (*Tagetes erecta* L.) sourced from three different regions. The aim was to identify the differential metabolites that play significant roles in differentiating marigold oleoresins based on their geographical origins. This research not only contributes to the scientific evaluation and optimal utilization of natural resources, but also enhances product quality and market competitiveness, fostering related scientific advancements, technological innovations, and sustainable agricultural development. In general, this research has profound scientific value and socio-economic significance.

## 2. Materials and Methods

### 2.1. Samples and Chemical Reagents

Raw materials of marigold granules were procured from the sales markets of their respective original production areas in March 2023: Zambia (FZ) in Africa, Xinjiang (XJ) in China, and India (YD). These materials were then utilized in subsequent oleoresin preparation experiments. For each origin, three batches were obtained and subjected to experimentation.

### 2.2. The Preparation of Marigold Oleoresin

After thorough mixing, 1.0 g of marigold granules was precisely weighed and transferred into a 100 mL volumetric flask. Next, *n*-hexane was added to the flask until it reached the calibration mark. Subsequently, the volumetric flask was immersed in a thermostatically controlled water bath maintained at 40 °C for extraction. At every 10 min interval, ultrasonication was performed for 5 min, and this process was repeated a total of four times. Following this, the flask was removed from the water bath and allowed to cool to room temperature. Then, once more, *n*-hexane was added up to the calibration mark. The resulting extract was then centrifuged at 12,000 rpm for 10 min at 4 °C. The supernatant was filtered through a 0.22 µm membrane into an autosampler vial and stored at 4 °C for subsequent mass spectrometry detection and analysis. This preparation procedure was identical for all batches of samples.

### 2.3. The Conditions of Chromatographic and Mass Spectrometry

A chromatographic analysis was performed using the ACQUITY UPLC I-class system, which is manufactured by Waters Corporation (Milford, MA, USA). This advanced analytical platform integrates a binary solvent management module, an automated sample injector, a vacuum degassing unit, and a temperature-controlled column chamber. For the chromatographic separations, an ACQUITY BEH C18 column (2.1 mm × 150 mm, 1.7 µm; Waters Corporation, Milford, MA, USA) was chosen. Employing a gradient elution strategy, the mobile phase was composed of two components: solvent A, containing 0.1% formic acid in deionized water, and solvent B, represented by a 1:1 volumetric blend of acetonitrile and isopropanol. The subsequent elution program was as follows: 0–2 min, 60%B; 2–50 min, 60–84%B; 50–120 min, 84–100%B; 120–121 min, 100%B; 121–122 min, 100–60%B; 122–124 min, 60%B. A sample injection volume of 3 µL was established, accompanied by a constant flow rate of 0.2 mL/min throughout the analysis. For mass spectrometric detection, a Waters Xevo G2-XS QTOF instrument, equipped with a Z Spray™ electrospray ionization (ESI) trap source, was employed. The desolvation gas flow rate was maintained at 800 L/h, while the cone gas flow rate was set to 50 L/h. The operating conditions for the ESI source included a source offset voltage of 80 V, a cone voltage of 40 V, and a source temperature of 100 °C. The capillary voltage was adjusted to 3.0 kV. Data acquisition proceeded under the Fast-DDA mode, with the dual dynamic collision energy ranging from 6 to 40 V for precursors up to 50 Da and from 40 to 120 V for those above 1500 Da. The MS scan rate was 0.04 s, whereas the MS/MS scan rate was 0.1 s. In the data-dependent acquisition, the top 3 most intense precursor ions underwent MS/MS fragmentation to generate the MN. Real-time calibration was achieved using leucine encephalin solution (*m*/*z* 556.2771 [M+H]^+^) as an internal reference. MassLynx v4.1 software (Waters Corporation, Milford, MA, USA) facilitated data acquisition.

### 2.4. Molecular Networking

The raw data from UPLC-QTOF-MS/MS were converted into “mzXML” format using MS Convert Version 3.0.23052-0c85f26 automated build (http://proteowizard.sourceforge.net, accessed on 17 May 2023) and subsequently uploaded to the GNPS online platform [15]. The following parameters were configured for GNPS analysis: a tolerance of 0.02 Da was applied to both the precursor ion masses and the fragment ion masses. A cosine score exceeding 0.7 with a minimum of six matched peaks was considered for the analysis. A molecular network was constructed, and its visualization was accomplished utilizing the Cytoscape software version 3.9.1, accessible at http://www.cytoscape.org, accessed on 17 May 2023.

### 2.5. Multivariate Statistical Analysis

The synergistic application of UPLC-QTOF-MS/MS technology and multivariate statistical analysis offers a comprehensive approach to characterizing chemical constituents and efficiently identifying potential chemical markers [16,17]. The raw mass spectrometry data were preprocessed using MSDIAL 4.92 (http://prime.psc.riken.jp/compms/msdial/main.html, accessed on 17 May 2023). Following preprocessing, the LC-MS data were subjected to multivariate statistical analysis within the SIMCA-P 14.1 software (MKS Umetrics AB, Sweden). To assess the relationships and differences among distinct experimental groups, a principal component analysis (PCA) and orthogonal partial least squares discriminant analysis (OPLS-DA) were conducted. PCA, an unsupervised technique, transforms the initial variables into a set of orthogonal, non-correlated components, thereby condensing redundant information. In contrast, OPLS-DA, which employs a supervised modeling technique, aims to eliminate systematic noise and selectively extract informative variables. Moreover, S-plots were generated to identify chemical markers by examining the Variable Importance for the Projection (VIP) values. A volcano plot analysis was performed using the MetaboAnalyst 5.0 web platform (https://www.metaboanalyst.ca, accessed on 17 May 2023), while a heatmap analysis was conducted using Hiplot (https://hiplot.cn/basic/heatmap, accessed on 17 May 2023).

## 3. Results and Discussion

### 3.1. Identifying Chemical Constituents in Marigold Oleoresins of Diverse Origins

**Figure 1:**
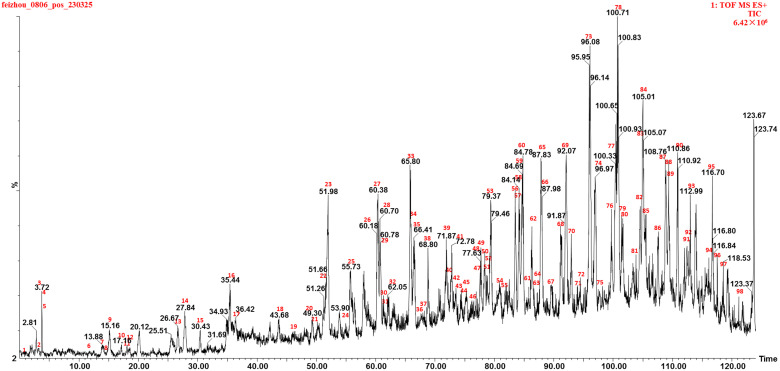
Representative base peak intensity chromatogram of marigold extract in the positive ion mode.

**Figure 2:**
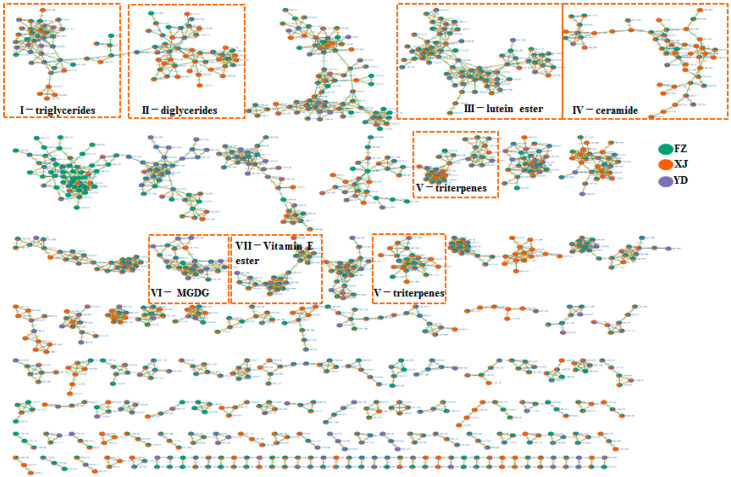
The molecular networks of marigold oleoresin with different regions. triglycerides (**I**), diglycerides (**II**), lutein esters (**III**), ceramide (**IV**), triterpenes(**V**), monogalactosyldiacylgylcerols (**VI**), and vitamin E esters (**VII**).

In this study, the comprehensive profiling of compounds in marigold oleoresin was achieved by integrating analytical data with a molecular networking chemical database and relevant literature resources. Consequently, a total of 83 compounds, including 6 Vitamin E and Vitamin E esters, 13 triterpenes, 7 lutein diesters, 4 lutein monoesters, 10 ceramide, 16 triglycerides, 13 diglycerides, 6 monogalactosyldiacylgylcerols, 3 steroids, and 2 others, were identified or tentatively characterized. The compounds were identified according to a well-established analytical methodology, which relied on comparing their retention times (Rt), fragment ions, and fragmentation pathways with corresponding data from the literature. A representative chromatogram of marigold oleoresin, displaying the base peak intensity in positive ion mode, can be seen in Figure 1. Additionally, Table 1 provides comprehensive details on the individual components, encompassing peak numbers, retention times (Rt), compound names, molecular formulas, classification, mass errors, and characteristic fragment ions. In this work, an integrative molecular network of marigold oleoresin samples from various regions was constructed based on the MS/MS spectral similarity of their constituents, as illustrated in Figure 2. This network comprised 2224 precursor ions, with 184 clusters (nodes consisting of at least 2 ions) and 991 single nodes. Further details regarding this molecular network can be accessed via the GNPS website (https://gnps-cytoscape.ucsd.edu/process?task=eafa6c21f8274b3390e634d651e1f8fd, accessed on 17 May 2023). In the MN, the relative content of a compound in the XJ, YD, and FZ samples is depicted by the proportions of the orange, purple, and green sectors, respectively, within each node. Molecular networking facilitates the analysis by structurally grouping related compounds into clusters, allowing for easier examination and comparison [18]. Molecular networking reveals that triglycerides (I), diglycerides (II), lutein esters (III), ceramides (IV), triterpenes (V), monogalactosyldiacylglycerols (VI), and vitamin E esters (VII) each form distinct clusters, indicative of their structural similarities within these respective classes. The compounds were identified using a well-established analytical approach that combined retention time (Rt) data, the GNPS library, and fragmentation pathways referenced from the literature.

### 3.2. Identify Elucidation of Triglycerides and Diglycerides

**Figure 3:**
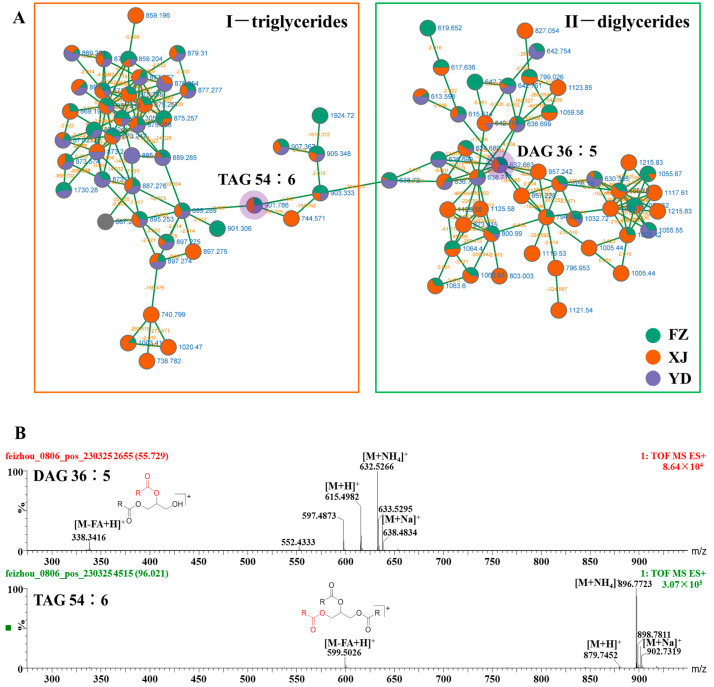
The enlargement of cluster I and II molecular networks (**A**), MS spectra (**B**) and possible fragmentation pathway of diglycerides 36:5 (DAG 36:5) and triglycerides 54:6 (TAG 54:6) from marigold oleoresin in positive ion mode.

Cluster I and Cluster II predominantly comprised nodes representing triglycerides and diglycerides, as evident in Figure 3. The primary observed adduct ions included [M+NH_4_]^+^, [M+Na]^+^, and [M+H]^+^. In the MS/MS spectra, a characteristic ion peak was generated by the loss of a single fatty acid fragment from triglycerides, represented as [M-FA+H]^+^ (Figure 2) [19,20]. The industrial production of marigold oleoresin yielded the identification of 16 triglycerides and 13 diglycerides.

### 3.3. Identify Elucidation of Lutein Esters

**Figure 4:**
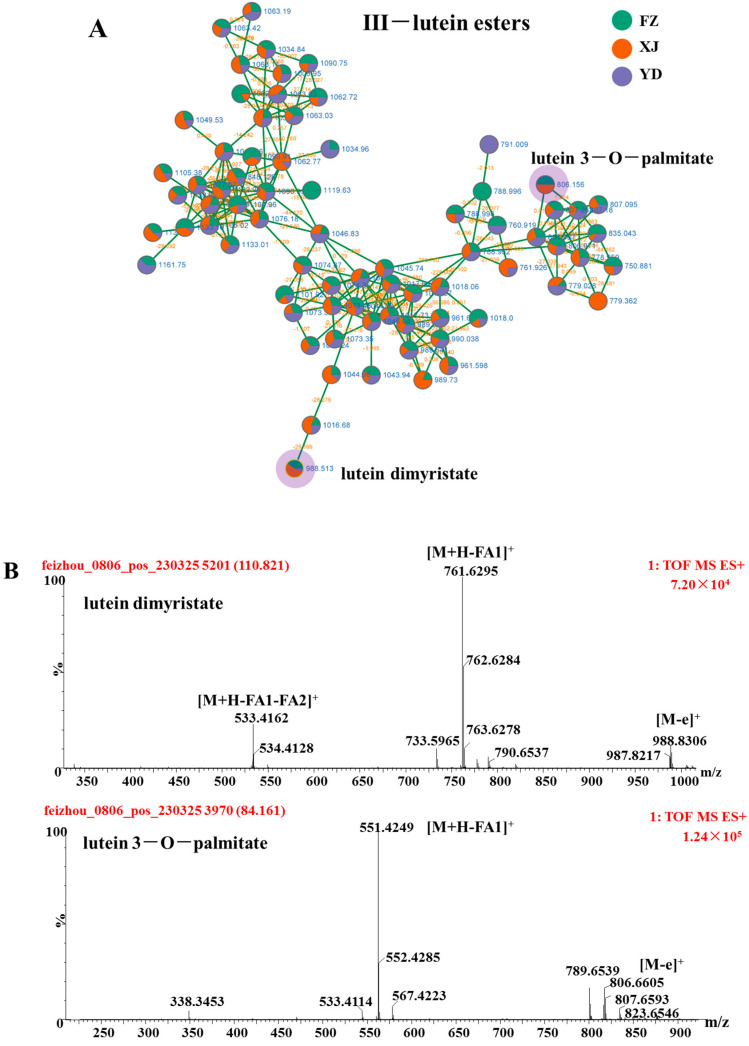

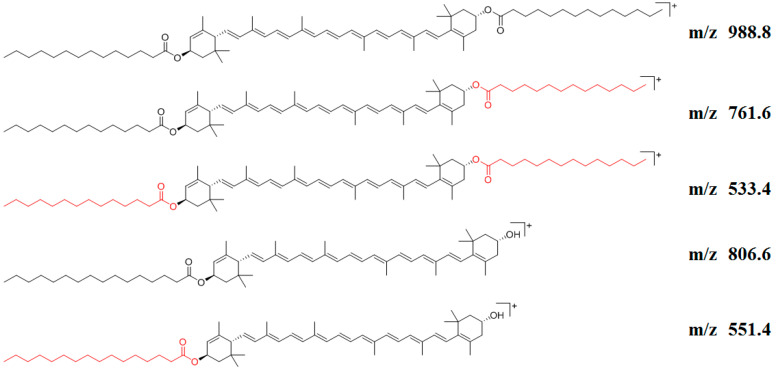
The enlargement of cluster III molecular network (**A**), MS spectra (**B**) and possible fragmentation pathway of lutein dimyristate and lutein 3-O-palmitate from marigold oleoresin in positive ion mode.

As displayed in Figure 4, Cluster III predominantly comprised nodes signifying lutein esters. The main components identified in marigold oleoresin were lutein esters, including lutein dilaurate, lutein laurate-myristate, lutein dimyristate, lutein myristate-palmitate, lutein dipalmitate, lutein palmitate-stearate, lutein distearate, lutein 3-O-laurate, lutein 3-O-myristate, lutein 3-O-palmitate, and lutein 3-O-stearate. These compounds exhibited characteristic mass fragmentation, with lutein monoesters showing a fragment ion at *m*/*z* 551.43 and lutein diesters showing a fragment ion at *m*/*z* 533.41 (Figure 3) [21,22,23,24,25].

### 3.4. Identify Elucidation of Other Compounds

The constituents within each cluster were tentatively classified as follows: Cluster IV represents putatively identified ceramides, Cluster V represents putatively identified triterpenes, Cluster VI represents putatively identified monogalactosyldiacylglycerols, and Cluster VII represents putatively identified vitamin E and vitamin E esters. The identification of these clusters was based on matching the fragment ions with those reported in the literature [26,27,28,29,30,31,32,33].

### 3.5. Differential Metabolomic Analysis of Marigold Oleoresin from Different Origins

#### PCA Analysis and Cluster Analysis

**Figure 5:**
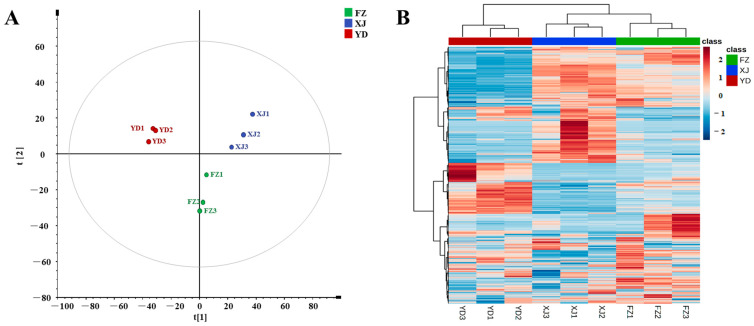
PCA score plot (**A**) and cluster analysis (**B**).

PCA analysis was performed on metabolites from three different origins of marigold oleoresin, as shown in Figure 5A. PC1 accounted for 43.9% of the total variance, while PC2 accounted for 20.6%. From the plot, it can be observed that samples from each group clustered together, indicating a clear separation trend among those from different origins. The PCA results overall reflected the differences in metabolites among the samples from different origins. A cluster analysis is illustrated in Figure 5B, which distinctly demonstrates distinct grouping patterns among the different origins. The metabolite types and contents of FZ and XJ were similar, clustering them together, while YD formed a separate cluster with significant differences from FZ and XJ. The combined PCA and cluster analysis indicated that the marigold oleoresin from the three different origins exhibited distinct metabolic characteristics.

**Figure 6:**
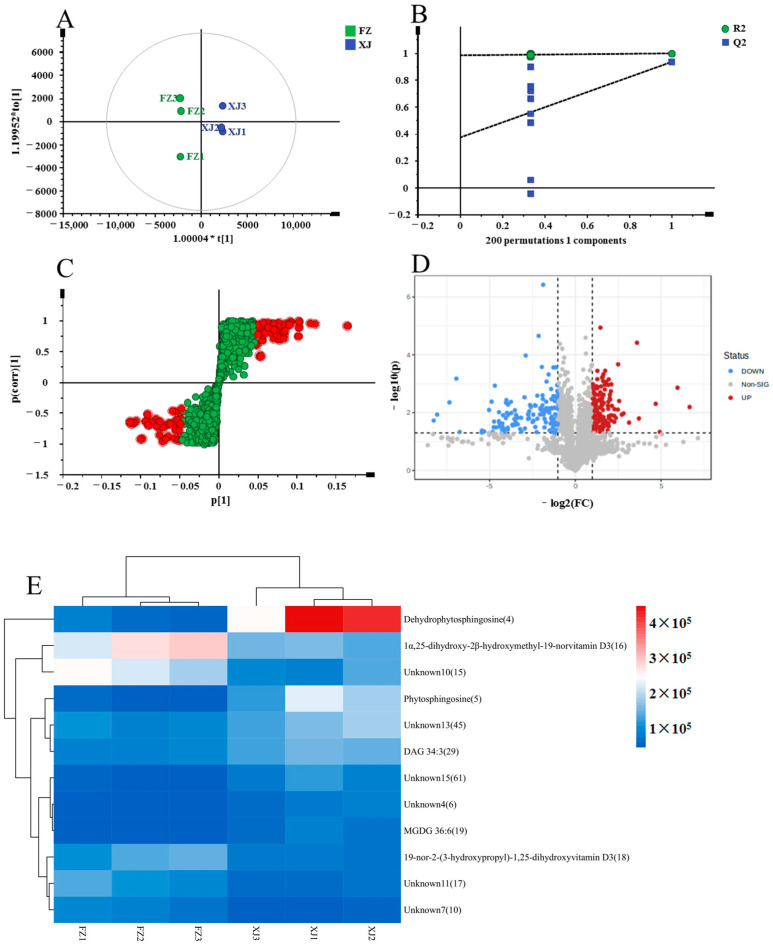
Multivariate statistical analysis result of FZ and XJ groups: OPLS-DA (**A**) score plots and OPLS-DA model permutation test (**B**), S-plot (**C**) and volcano plots (**D**), (**C**,**D**) were used to explore markers in FZ and XJ, the heatmap of potential chemical markers (**E**).

In conducting a pairwise comparison between FZ and XJ utilizing the statistical method OPLS-DA, several parameters were evaluated to assess the model’s performance. The R^2^X (cum) value, which indicates the explained variation in X (predictor) variables, was found to be 0.845. The R^2^Y (cum) value, representing the explained variation in Y (response) variables, was 1, indicating a good fit of the model (Figure 6A). The Q^2^ (cum) value, which estimates the model’s predictive ability, was determined to be 0.939, suggesting a high predictability. To validate the model, a permutation test was conducted with 200 iterations. The permutation plot showed that both the Q^2^ and R^2^ values obtained from the permutations (R^2^ = [0.0, 0.984]; Q^2^ = [0.0, 0.375]) were significantly lower than the original values (Figure 6B). This indicates that the model did not exhibit randomness and overfitting, further supporting its reliability. S-plots and volcano plots (Figure 6C,D) were generated under the OPLS-DA model to facilitate the rapid and visual identification of key markers. In S-plots, red indicates a value of VIP > 2, while green signifies VIP < 2. In the volcano plots, red spots represent variables with significant differences and a high abundance, while green spots represent variables with significant differences but a low abundance. Chemical markers that met certain criteria (VIP > 2, FC > 2 or <0.5, and *p* < 0.05) in the univariate statistical analysis were considered to be significant. Twelve markers displaying notable differences between FZ and XJ were identified and are listed in Table 2. These potential markers were further visualized using a heatmap (Figure 6E). In the heatmap, rows correspond to individual metabolites, while columns represent distinct samples. The coloration of each cell within the heatmap reflects the expression level of the respective metabolite in the corresponding sample. In the heatmap, high metabolite concentrations are denoted by red, whereas low concentrations are represented by blue. From the heatmap, it can be observed that one chemical marker (peak 4) exhibited higher relative contents in XJ compared to FZ. On the other hand, two chemical markers (peak 15,16) were found to be higher in FZ than in XJ. These markers represented metabolites that are potentially important in distinguishing between FZ and XJ.

**Figure 7:**
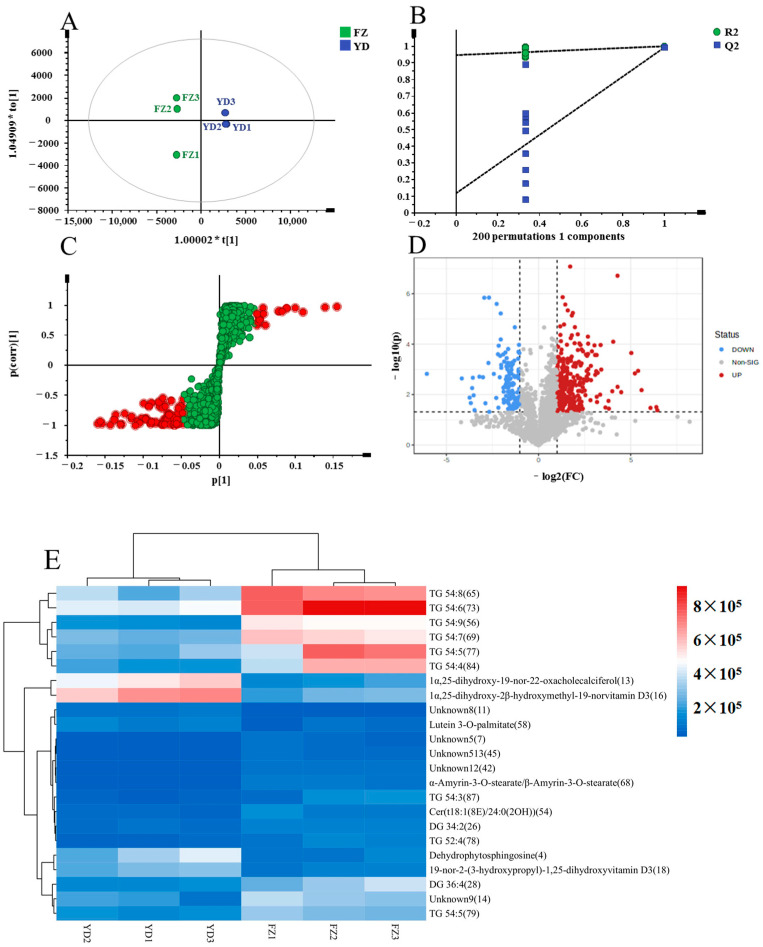
Multivariate statistical analysis result of FZ and YD groups: OPLS-DA (**A**) score plots and OPLS-DA model permutation test (**B**), S-plot (**C**) and volcano plots (**D**), (**C**,**D**) were used to explore markers in FZ and YD, the heatmap of potential chemical markers (**E**).

Similarly, a pairwise comparison was conducted between the FZ and YD groups. The OPLS-DA score plot further confirmed a significant distinction between the two groups, indicating a highly significant model (R^2^X = 0.889, R^2^Y = 1, Q^2^ = 0.993) (Figure 7A). The established OPLS-DA model (Figure 7B) demonstrated reliability and reproducibility (R^2^ = [0.0, 0.948], Q^2^ = [0.0, 0.119]). Additionally, S-plots and volcano plots (depicted in Figure 7C,D) were generated in order to pinpoint potential chemical markers. By applying criteria such as VIP > 2, FC > 2 or <0.5, and *p* < 0.05, a total of 23 potential markers were identified, as presented in Table 3. The results were visually represented in a heatmap format (Figure 7E) for intuitive interpretation. Furthermore, it was observed that FZ exhibited significantly higher levels of glycerolipids, including TG 54:8 (65), TG 54:6 (73), TG 54:9 (56), TG 54:7 (69), TG 54:5 (77), and TG 54:4 (84), compared to YD. Conversely, the steroids 1α,25-dihydroxy-19-nor-22-oxacholecalciferol (13) and 1α,25-dihydroxy-2β-hydroxymethyl-19-norvitamin D3 (16) had higher contents in YD compared to FZ.

**Figure 8:**
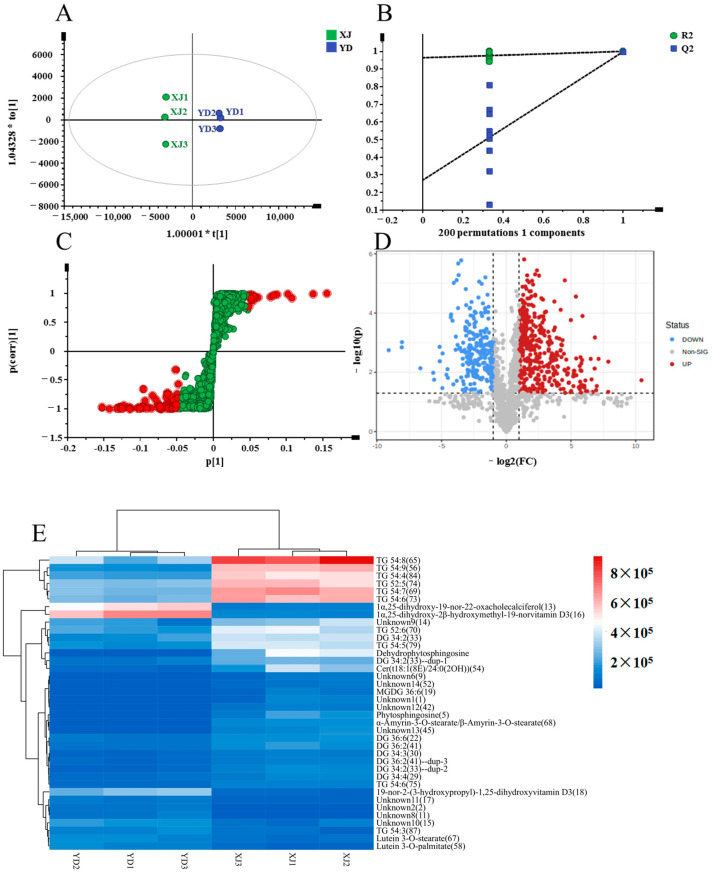
Multivariate statistical analysis result of XJ and YD groups: OPLS-DA (**A**) score plots and OPLS-DA model permutation test (**B**), S-plot (**C**) and volcano plots (**D**), (**C**,**D**) were used to explore markers in XJ and YD, the heatmap of potential chemical markers (**E**).

Likewise, a comparative study was executed between the XJ and YD samples. The OPLS-DA score plot confirmed a significant distinction between the two groups, indicating a highly significant model (R^2^X = 0.905, R^2^Y = 1, Q^2^ = 0.997) (Figure 8A). The established OPLS-DA model (Figure 8B) demonstrated reliability and reproducibility (R^2^ = [0.0, 0.965], Q^2^ = [0.0, 0.27]). S-plots and volcano plots (Figure 8C,D) were generated to identify potential chemical markers. By applying criteria such as VIP > 2, FC > 2 or < 0.5, and *p* < 0.05, a total of 39 potential markers were identified and are presented in Table 4. The results were visually represented in a heatmap format (Figure 7E) for intuitive interpretation. Moreover, XJ exhibited significantly higher levels of glycerolipids, including TG 54:8 (65), TG 54:6 (73), TG 54:9 (56), TG 54:7 (69), TG 54:5 (77), and TG 54:9 (84), compared to YD. Conversely, YD showed a higher sterol content, specifically in 1α,25-dihydroxy-19-nor-22-oxacholecalciferol (13) and 1α,25-dihydroxy-2β-hydroxymethyl-19-norvitamin D3 (16), compared to XJ.

In conclusion, the secondary metabolite composition of marigold flowers is significantly influenced by variations in soil conditions, climatic factors, and growth environments specific to their regional origins. As evidenced by the classification of the differential metabolites identified in samples from three distinct production areas, these variations predominantly involve non-pigment components. The relatively small differences observed in pigment content among the samples from the three regions indicate that pigments are not only influenced by environmental factors, but also have inherent associations with factors such as variety, cultivation management, harvesting, processing, and quality control. In industrial applications, marigold flowers, serving as a source of pigments, demonstrate a limited impact of production region on their pigment content, thus contributing to the stability of supply and consistency in product quality. Moreover, non-pigment components, particularly glycerides, sterols, and steroid compounds, are significantly affected by regional environmental factors. From a scientific perspective, this diversity facilitates the elucidation of connections between non-pigment constituents in marigold flowers and ecological factors, genetic determinants, and so on, spurring advancements in fields such as plant chemistry, ecology, and genetic breeding. Concurrently, this enables the development of diversified products tailored to market demands and customer preferences. Additionally, the analytical methodology established in this study is not only applicable to the comparative analysis of marigold oil resin samples from different production regions, but also possesses the versatility to be extended to the investigation of variations among samples derived from various plant species and distinct processing techniques.

## 4. Conclusions

A comprehensive approach combining a multi-dimensional sampling strategy and diverse data analysis techniques was devised to characterize and classify marigold oleoresin samples originating from three different regions. Using GNPS, a total of 83 compounds were identified or tentatively characterized in the marigold oleoresin samples. These constituents included 6 vitamin E and vitamin E esters, 13 triterpenes, 7 lutein diesters, 4 lutein monoesters, 10 ceramides, 16 triglycerides, 13 diglycerides, 6 monogalactosyldiacylglycerols, 3 steroids, and 2 others. Subsequently, a multivariate statistical analysis coupled with a heatmap revealed clear distinctions among the marigold oleoresin samples from the three different regions. Specifically, a total of 12, 23, and 38 significantly different metabolites were identified between FZ and XJ, FZ and YD, and XJ and YD, respectively. This finding indicates significant variations in the metabolite composition of marigold oleoresin based on its origin. The results of this study can be used to distinguish marigold oleoresin samples from different regions, laying a solid foundation for further quality control and providing a theoretical basis for assessing safety and nutritional aspects.

## Figures and Tables

**Table 1:** Identification of the chemical compounds of marigold oleoresin by UPLC-QTOF-MS.

No.	Rt	Adduct Mode	Identification	Formula	MS	Error	MS/MS	Classification
1	1.89	[M+H]^+^	Unknown 1		469.4150			
2	3.21	[M+H]^+^	Unknown 2		219.1035			
3	3.72	[M+H]^+^	Unknown 3		277.2181			
4	3.72	[M+H]^+^	Dehydrophytosphingosine	C_18_H_37_NO_3_	316.2857	1.6	298.2753; 280.2383; 262.2272	Sphingoids
5	3.74	[M+H]^+^	Phytosphingosine	C_18_H_39_NO_3_	318.2992	−5.0	300.291; 256.2637	Sphingoids
6	11.26	[M+H]^+^	Unknown 4		384.3506			
7	13.88	[M+Na]^+^	Unknown 5		361.2690		321.2783; 261.2186	
8	14.20	[M+H]^+^	Erythrodiol/Uvaol	C_30_H_50_O_3_	443.3912	5.2	425.3768; 407.3655; 235.2063; 217.1955; 203.1786; 191.1808	Triterpenes
9	15.18	[M+H]^+^	Unknown 6		593.2728			
10	17.17	[M+H]^+^	Unknown 7		323.2970			
11	18.17	[M+H]^+^	Unknown 8		379.2860			
12	18.48	[M+H]^+^	α-Amyrin/β-Amyrin	C_30_H_50_O	427.3934	−1.4	409.3831; 271.2409; 217.1966; 191.1809; 149.1318; 135.1171	Triterpenes
13	26.70	[M+H]^+^	1α,25-dihydroxy-19-nor-22-oxacholecalciferol	C_25_H_42_O_4_	407.3151	−2.5	179.1062	Steroid
14	27.80	[M+H]^+^	Unknown 9		351.2918			
15	30.42	[M+H]^+^	Unknown 10		321.2783			
16	35.50	[M+H]^+^	1α,25-dihydroxy-2β-hydroxymethyl-19-norvitamin D3	C_25_H_46_O_4_	435.3490	4.1	179.1062	Steroid
17	36.38	[M+H]^+^	Unknown 11		323.2928			
18	43.68	[M+H]^+^	19-nor-2-(3-hydroxypropyl)-1,25-dihydroxyvitamin D3	C_29_H_50_O_4_	463.3810	3.9	179.1062	Steroid
19	46.42	[M+Na]^+^	MGDG 36:6	C_45_H_74_O_10_	797.5176	−0.5	613.4835; 519.2935; 335.2567	Monogalactosyldiacylgylcerols
20	49.27	[M+Na]^+^	MGDG 36:5	C_45_H_76_O_10_	799.5338	0.3	615.5005; 597.4919; 337.2736	Monogalactosyldiacylgylcerols
21	49.30	[M-e]^+^	β-Tocopherol/ϒ-Tocopherol	C_28_H_48_O_2_	416.3636	−2.6	205.1214; 165.0912	Vitamin E
22	51.62	[M+Na]^+^	DAG 36:6	C_39_H_64_O_5_	635.4658	1.1	630.5075	Diglycerides
23	51.98	[M-e]^+^	α-Tocopherol	C_29_H_50_O_2_	430.3791	5.3	205.1243; 165.0912	Vitamin E
24	55.10	[M+Na]^+^	MGDG 36:4	C_45_H_78_O_10_	801.5525	−3.2	617.5208; 599.5074; 337.2774	Monogalactosyldiacylgylcerols
25	55.76	[M+Na]^+^	DAG 36:5	C_39_H_66_O_5_	637.4824	2.5	632.5266	Diglycerides
26	60.18	[M+Na]^+^	MGDG 34:2	C_43_H_78_O_10_	777.5471	−2.8	575.5054; 521.3080; 337.2736	Monogalactosyldiacylgylcerols
27	60.31	[M+Na]^+^	DAG 36:4	C_39_H_68_O_5_	639.4971	1.1	634.5436	Diglycerides
28	60.75	[M+Na]^+^	DAG 36:4	C_39_H_68_O_5_	639.4971	1.1	634.5436	Diglycerides
29	60.82	[M+Na]^+^	DAG 34:3	C_37_H_66_O_5_	613.4835	4.4	608.5262	Diglycerides
30	61.31	[M+Na]^+^	DAG 34:3	C_37_H_66_O_5_	613.4835	4.4	608.526	Diglycerides
31	61.52	[M+Na]^+^	Primulagenin A-3-O-myristate	C_44_H_76_O_4_	691.5630	−1.6	651.5735; 423.3634; 405.3535	Triterpenes
32	62.03	[M+Na]^+^	MGDG 36:3	C_45_H_80_O_10_	803.5680	3.9	601.5214; 519.2935; 341.3067	Monogalactosyldiacylgylcerols
33	65.84	[M+Na]^+^	DAG 34:2	C_37_H_68_O_5_	615.4954	−1.6	610.54	Diglycerides
34	66.02	[M+Na]^+^	DAG 34:2	C_37_H_68_O_5_	615.4954	−1.6	610.54	Diglycerides
35	66.46	[M+Na]^+^	DAG 34:2	C_37_H_68_O_5_	615.4954	−1.6	610.54	Diglycerides
36	67.12	[M+Na]^+^	MGDG 36:2	C_45_H_82_O_10_	805.5860	−0.5	603.5387; 521.3127; 341.3067	Monogalactosyldiacylgylcerols
37	68.20	[M+Na]^+^	DAG 36:3	C_39_H_70_O_5_	641.5149	2.8	636.5588	Diglycerides
38	68.80	[M+Na]^+^	Primulagenin A-3-O-palmitate	C_46_H_80_O_4_	719.5989	4.9	679.6047; 423.3634; 405.3535	Triterpenes
39	72.02	[M+H]^+^	Erythrodiol-3-O-myristate	C_44_H_76_O_3_	653.5862	−1.7	425.3764; 407.3687	Triterpenes
40	72.17	[M-e]^+^	Lutein 3-O-laurate	C_52_H_78_O_3_	750.5967	4.4	733.6458; 551.5039	Lutein esters
41	72.79	[M+Na]^+^	DAG 36:2	C_39_H_72_O_5_	643.5256	−3.3	638.572	Diglycerides
42	73.11	[M+H]^+^	Unknown12		871.5735			
43	73.48	[M+Na]^+^	Cer(t18:1(8E)/22:0(2OH))	C_40_H_79_NO_5_	676.5863	1	654.6037; 636.5949; 298.2756; 280.2632	Ceramide
44	73.50	[M+Na]^+^	DAG 36:2	C_39_H_72_O_5_	643.5256	−3.3	638.572	Diglycerides
45	74.41	[M+NH_4_]^+^	Unknown 13		1052.7786			
46	76.12	[M+Na]^+^	Primulagenin A-3-O-stearate	C_48_H_84_O_4_	747.6255	−1.6	707.6334; 423.3634; 405.3535	Triterpenes
47	77.15	[M+Na]^+^	Cer(t18:1(8E)/23:0(2OH))	C_41_H_81_NO_5_	690.6064	−0.1	668.6174; 650.6085; 298.2756; 280.2632	Ceramide
48	77.71	[M+H]^+^	α-Amyrin-3-O-myristate/β-Amyrin-3-O-myristate	C_44_H_76_O_2_	637.5908	−2.5	409.3849	Triterpenes
49	77.76	[M+Na]^+^	Cer(t18:1/22:0(2OH))	C_40_H_81_NO_5_	678.6031	2.8	656.6243; 638.6133; 300.2916; 282.2792	Ceramide
50	78.35	[M-e]^+^	Lutein 3-O-myristate	C_54_H_82_O_3_	778.6249	−1.9	761.6295; 551.4249	Lutein esters
51	78.62	[M+Na]^+^	DAG 36:1	C_39_H_74_O_5_	645.5446	1.9	640.5873	Diglycerides
52	78.96	[M+NH_4_]^+^	Unknown 14		1054.7958			
53	79.37	[M+H]^+^	Erythrodiol-3-O-palmitate	C_46_H_80_O_3_	681.6185	−0.1	425.3764; 407.3687	Triterpenes
54	80.66	[M+Na]^+^	Cer(t18:1(8E)/24:0(2OH))	C_42_H_83_NO_5_	704.6184	2.1	682.6353; 664.6270; 298.2756; 280.2632	Ceramide
55	81.07	[M+Na]^+^	Cer(t18:1/23:0(2OH))	C_41_H_83_NO_5_	692.6172	0.4	670.6330; 652.6281; 300.2916; 282.2792	Ceramide
56	83.54	[M+Na]^+^	TG 54:9	C_57_H_92_O_6_	895.6830	4.2	890.7225	Triglycerides
57	83.96	[M+Na]^+^	Cer(t18:1(8E)/25:0(2OH))	C_43_H_84_NO_5_	718.6340	−5.4	696.6529; 678.6404; 298.2756; 280.2632	Ceramide
58	84.14	[M-e]^+^	Lutein 3-O-palmitate	C_56_H_86_O_3_	806.6540	−4.6	789.6539; 551.4249	Lutein esters
59	84.58	[M+Na]^+^	Cer(t18:1/24:0(2OH))	C_42_H_85_NO_5_	706.6385	0.8	684.6558; 666.6418; 300.2916; 282.2792	Ceramide
60	84.76	[M+H]^+^	α-Amyrin-3-O-palmitate/β-Amyrin-3-O-palmitate	C_46_H_80_O_2_	665.6235	−0.3	409.3849	Triterpenes
61	85.43	[M+NH_4_]^+^	Unknown 15		856.7020			
62	86.28	[M+H]^+^	Erythrodiol-3-O-stearate	C_48_H_84_O_3_	709.6470	1.8	425.3764; 407.3687	Triterpenes
63	87.12	[M+Na]^+^	Cer(t18:1/25:0(2OH))	C_43_H_87_NO_5_	720.6468	−1.9	698.6671; 680.6604; 300.2916; 282.2792	Ceramide
64	87.32	[M+Na]^+^	Cer(t18:1(8E)/26:0(2OH))	C_44_H_87_NO_5_	732.6494	1.6	710.6713; 692.6602; 298.2756; 280.2632	Ceramide
65	87.87	[M+Na]^+^	TG 54:8	C_57_H_94_O_6_	897.6968	2.2	892.7369	Triglycerides
66	88.23	[M+H]^+^	β-Tocopherol dodecanoate	C_40_H_70_O_3_	599.5376	−0.5	417.3743	Vitamin E esters
67	89.56	[M-e]^+^	Lutein 3-O-stearate	C_58_H_90_O_3_	834.6907	2	817.6877; 551.4249	Lutein esters
68	91.18	[M+H]^+^	α-Amyrin-3-O-stearate/β-Amyrin-3-O-stearate	C_48_H_84_O_2_	693.6560	1.4	409.3849	Triterpenes
69	92.09	[M+Na]^+^	TG 54:7	C_57_H_96_O_6_	899.7128	2.6	894.7534	Triglycerides
70	92.92	[M+Na]^+^	TG 52:6	C_55_H_94_O_6_	873.6989	−2.2	868.7396	Triglycerides
71	94.03	[M+Na]^+^	Cer(t18:1/26:0(2OH))	C_44_H_89_NO_5_	734.6650	−5.9	712.6837; 694.6740; 300.2916; 282.2792	Ceramide
72	94.26	[M+H]^+^	β-Tocopherol myristate	C_42_H_74_O_3_	627.5754	6.1	417.3743	Vitamin E esters
73	96.10	[M+Na]^+^	TG 54:6	C_57_H_98_O_6_	901.7271	1.1	896.7736	Triglycerides
74	96.99	[M+Na]^+^	TG 52:5	C_55_H_96_O_6_	875.7120	1.7	870.7529	Triglycerides
75	97.76	[M+Na]^+^	TG 54:6	C_57_H_98_O_6_	901.7271	1.1	896.7736	Triglycerides
76	99.78	[M+H]^+^	β-Tocopherol palmitate	C_44_H_76_O_3_	655.5997	−4.9	417.3701	Vitamin E esters
77	100.43	[M+Na]^+^	TG 54:5	C_57_H_100_O_6_	903.7418	0	898.7889	Triglycerides
78	100.77	[M+Na]^+^	TG 52:4	C_55_H_98_O_6_	877.7259	−0.2	872.7724	Triglycerides
79	101.50	[M+Na]^+^	TG 54:5	C_57_H_100_O_6_	903.7418	0	898.7889	Triglycerides
80	101.71	[M+Na]^+^	TG 50:3	C_53_H_96_O_6_	851.7140	−2.9	846.7572	Triglycerides
81	103.95	[M+Na]^+^	Lutein dilaurate	C_64_H_100_O_4_	955.7567	5	932.7582; 733.5905; 533.4156	Lutein esters
82	104.66	[M+H]^+^	β-Tocopherol stearate	C_46_H_78_O_3_	683.6373	4.5	417.3659	Vitamin E esters
83	104.88	[M+Na]^+^	TG 52:3	C_55_H_100_O_6_	879.7440	2.5	874.7908	Triglycerides
84	105.08	[M+Na]^+^	TG 54:4	C_57_H_102_O_6_	905.7588	1.5	900.8004	Triglycerides
85	105.36	[M+Na]^+^	TG 50:2	C_53_H_98_O_6_	853.7318	6.7	848.7751	Triglycerides
86	107.60	[M+Na]^+^	Lutein laurate-myristate	C_66_H_104_O_4_	983.7872	4.1	960.7942; 761.6221; 733.5905; 533.4156	Lutein esters
87	108.90	[M+Na]^+^	TG 54:3	C_57_H_104_O_6_	907.7719	−1.3	902.8202	Triglycerides
88	109.32	[M+Na]^+^	TG 52:2	C_55_H_102_O_6_	881.7582	0.9	876.7994	Triglycerides
89	109.34	[M+Na]^+^	TG 50:1	C_53_H_100_O_6_	855.7460	4.9	850.7894	Triglycerides
90	111.33	[M+Na]^+^	Lutein dimyristate	C_68_H_108_O_4_	1011.8197	5.1	988.8144; 761.6277; 533.4156	Lutein esters
91	112.60	[M+Na]^+^	Lutein myristate-palmitate	C_70_H_112_O_4_	1039.8439	−1.8	1016.8593; 789.6564; 761.6221; 533.4156	Lutein esters
92	112.91	[M+Na]^+^	TG 54:2	C_57_H_106_O_6_	909.7933	−1.8	904.8361	Triglycerides
93	112.99	[M+Na]^+^	TG 52:1	C_55_H_104_O_6_	883.7748	1.9	878.8225	Triglycerides
94	116.31	[M+Na]^+^	TG 54:1	C_57_H_108_O_6_	911.8047	7.1	906.8543	Triglycerides
95	116.62	[M+Na]^+^	TG 56:2	C_59_H_110_O_6_	937.8226	2.8	932.8644	Triglycerides
96	117.14	[M+Na]^+^	Lutein dipalmitate	C_72_H_116_O_4_	1067.8833	5.8	1044.8865; 789.6564; 533.4156	Lutein esters
97	118.53	[M+Na]^+^	Lutein palmitate-stearate	C_74_H_120_O_4_	1095.9099	1.4	1072.9198; 817.6887; 789.6505; 533.4156	Lutein esters
98	121.17	[M+Na]^+^	Lutein distearate	C_76_H_124_O_4_	1123.9432	3.1	1100.9509; 817.6829; 533.4109	Lutein esters

**Table 2:** Statistical table of specific differential metabolites of FZ vs. XJ.

No.	Compound Name	VIP Value	Fold Change	*p*-Value
1	Dehydrophytosphingosine (4)	6.93	0.09	0.0083
2	Phytosphingosine (5)	4.34	0.09	0.0166
3	1α,25-dihydroxy-2β-hydroxymethyl-19-norvitamin D3 (16)	4.22	2.06	0.0089
4	Unknown 10 (15)	4.13	2.63	0.0136
5	Unknown 13 (45)	2.98	0.48	0.0252
6	DAG 34:3 (29)	2.91	0.49	0.0032
7	19-nor-2-(3-hydroxypropyl)-1,25-dihydroxyvitamin D3 (18)	2.89	2.56	0.0105
8	Unknown 11 (17)	2.73	3.06	0.0252
9	Unknown 15 (61)	2.65	0.15	0.0157
10	Unknown 7 (10)	2.42	6.55	0.0117
11	Unknown 4 (6)	2.30	0.07	0.0120
12	MGDG 36:6 (19)	2.12	0.09	0.0121

**Table 3:** Statistical table of specific differential metabolites of FZ vs. YD.

No.	Compound Name	VIP Value	Fold Change	*p*-Value
1	Dehydrophytosphingosine (4)	4.65	0.28	0.0171
2	Unknown 5 (7)	2.04	4.48	0.0166
3	Unknown 8 (11)	2.42	0.23	0.0021
4	1α,25-dihydroxy-19-nor-22-oxacholecalciferol (13)	5.90	0.33	0.0012
5	Unknown 9 (14)	4.06	2.26	0.0204
6	1α,25-dihydroxy-2β-hydroxymethyl-19-norvitamin D3 (16)	6.54	0.36	0.0007
7	19-nor-2-(3-hydroxypropyl)-1,25-dihydroxyvitamin D3 (18)	4.19	0.35	0.0023
8	DG 36:4 (28)	4.09	2.34	0.0168
9	DG 34:2 (26)	2.31	2.05	0.0022
10	Unknown 12 (42)	2.49	10.32	0.0001
11	Unknown 13 (45)	2.39	10.57	0.0010
12	Cer (t18:1(8E)/24:0(2OH)) (54)	2.53	2.71	0.0235
13	TG 54:9 (56)	6.04	3.50	0.0000
14	Lutein 3-O-palmitate (58)	2.46	0.37	0.0282
15	TG 54:8 (65)	6.51	2.36	0.0017
16	α-amyryl octadecanoate/β-octadecanoate (68)	2.77	8.37	0.0003
17	TG 54:7 (69)	5.51	2.15	0.0003
18	TG 54:6 (73)	6.75	2.04	0.0011
19	TG 54:5 (77)	5.74	2.51	0.0418
20	TG 54:4 (78)	2.45	2.57	0.0065
21	TG 54:5 (79)	3.81	2.03	0.0016
22	TG 54:4 (84)	5.85	3.02	0.0131
23	TG 54:3 (87)	2.80	4.81	0.0445

**Table 4:** Statistical table of specific differential metabolites of XJ vs. YD.

No.	Compound Name	VIP Value	Fold Change	*p*-Value
1	Unknown 2 (2)	2.15	0.12	0.0006
2	Dehydrophytosphingosine (4)	5.15	51.10	0.0062
3	Phytosphingosine (5)	3.21	37.38	0.0133
4	Unknown 10 (15)	2.58	0.45	0.0063
5	Unknown 11 (17)	2.03	0.33	0.0012
6	Unknown 9 (14)	3.32	2.13	0.0371
7	Unknown 8 (11)	2.26	0.15	0.0012
8	1α,25-dihydroxy-19-nor-22-oxacholecalciferol (13)	5.75	0.18	0.0003
9	1α,25-dihydroxy-2β-hydroxymethyl-19-norvitamin D3 (16)	6.55	0.18	0.0002
10	19-nor-2-(3-hydroxypropyl)-1,25-dihydroxyvitamin D3 (18)	4.29	0.14	0.0005
11	Unknown 1 (1)	2.35	57.87	0.0270
12	Lutein 3-O-stearate (67)	2.18	0.47	0.0152
13	DG 34:4(29)	2.05	2.07	0.0059
14	Unknown 6 (9)	2.05	11.51	0.0268
15	DG 34:3 (30)	2.04	2.55	0.0012
16	DG 34:2 (33)	4.12	2.44	0.0006
17	DG 34:2 (33)	3.57	3.20	0.0001
18	DG 34:2 (33)	2.59	3.63	0.0011
19	DG 36:6 (22)	2.45	2.07	0.0001
20	DG 36:2 (41)	2.56	2.32	0.0011
21	DG 36:2 (41)	2.02	2.27	0.0019
22	Cer(t18:1(8E)/24:0(2OH)) (54)	3.95	6.48	0.0266
23	α-amyryl octadecanoate/β-octadecanoate (68)	3.07	12.77	0.0004
24	Lutein 3-O-palmitate (58)	2.40	0.32	0.0068
25	MGDG 36:6 (19)	2.04	101.92	0.0469
26	TG 52:6 (70)	4.08	2.22	0.0020
27	Unknown 12 (42)	2.39	12.67	0.0040
28	TG 52:5 (74)	4.78	2.04	0.0002
29	TG 54:9 (56)	5.98	4.18	0.0000
30	TG 54:8 (65)	6.43	2.72	0.0011
31	TG 54:7 (69)	5.57	2.52	0.0001
32	TG 54:6 (75)	2.17	2.39	0.0004
33	TG 54:6 (73)	5.37	2.41	0.0001
34	TG 54:5 (79)	4.38	2.72	0.0000
35	TG 54:4 (84)	5.24	2.91	0.0001
36	TG 54:3 (87)	2.18	0.45	0.0092
37	Unknown 13 (45)	3.07	21.85	0.0024
38	Unknown 14 (52)	2.15	23.50	0.0088

## Data Availability

The original contributions presented in the study are included in the article, further inquiries can be directed to the corresponding author.
